# N^6^-methyladenosine-modified lncRNA and mRNA modification profiles in cerebral ischemia-reperfusion injury

**DOI:** 10.3389/fgene.2022.973979

**Published:** 2022-11-21

**Authors:** Le Shao, Bowei Chen, Qibiao Wu, Yaqian Xu, Jian Yi, Zhihua Guo, Baiyan Liu

**Affiliations:** ^1^ The First Hospital, Hunan University of Chinese Medicine, Changsha, China; ^2^ MOE Key Laboratory of Research & Translation on Prevention & Treatment of Major Diseases in Internal Medicine of Traditional Chinese Medicine, Changsha, China; ^3^ Faculty of Chinese Medicine and State Key Laboratory of Quality Research in Chinese Medicine, Macau University of Science and Technology, Taipa, Macao SAR, China; ^4^ Zhuhai MUST Science and Technology Research Institute, Zhuhai, China; ^5^ College of Chinese Medicine, Hunan University of Chinese Medicine, Changsha, China; ^6^ Hunan Academy of Chinese Medicine, Changsha, China

**Keywords:** cerebral ischemia-reperfusion injury, N^6^-methyladenosine, lncRNA, mRNA, FTO

## Abstract

Cerebral ischemia-reperfusion injury (CIRI) is common in ischemic stroke and seriously affects the prognosis of patients. At present, N^6^-methyladenosine (m^6^A) modification of lncRNAs and mRNAs has been reported in other diseases, such as cancer, but its role in CIRI has not been clarified. In this study, we aimed to investigate the m^6^A lncRNA and m^6^A mRNA modification profiles in CIRI. First, we detected the total level of m^6^A and the changes in related m^6^A methyltransferases and demethylases in the brain tissue of rats with CIRI and then identified differentially modified lncRNAs and mRNAs in CIRI by lncRNA and mRNA epigenetic transcriptomic microarray. In addition, bioinformatics analysis was used to predict the underlying functions and related pathways of related lncRNAs and mRNAs. We found that the total m^6^A methylation level was significantly increased, and the expression of fat mass and obesity-associated protein (*FTO*) was downregulated after CIRI. In addition, a large number of m^6^A-modified lncRNAs and mRNAs appeared after CIRI, and these genes were mainly enriched for the Toll-like receptor signaling pathway, peroxisome proliferator-activated receptor (PPAR) signaling pathway, and mitogen-activated protein kinase (MAPK) signaling pathway. Our findings provide the basis and insights for further studies on m^6^A modification in CIRI.

## Introduction

The primary treatment principle for ischemic stroke is to recanalize the blood flow in the ischemic area and restore the blood oxygen supply to the infarcted brain tissue as soon as possible, but it may cause cerebral ischemia-reperfusion injury (CIRI) and secondary injury to the brain tissue (van der Steen et al., 2022). The mechanism underlying CIRI is very complex and may be related to oxidative stress, calcium overload, inflammation, *etc.* (Stegner et al., 2019), which eventually lead to nerve cell damage, apoptosis, or necrosis. Further exploration of the pathogenesis and prognostic biomarkers of CIRI or identification of therapeutic targets may be of great significance.

Long noncoding RNAs (lncRNAs) are a class of RNAs that cannot encode translational proteins but can actively participate in many important biological processes by regulating gene expression at the transcriptional and posttranscriptional levels. In recent years, studies have shown that lncRNAs are involved in pathophysiological responses such as inflammation, oxidative stress, angiogenesis, and nerve regeneration after cerebral ischemia (Akella et al., 2019). Currently, the number of studies involving related lncRNAs involved in CIRI is increasing, and a recent study reported lncRNA expression profiles after CIRI (Yang et al., 2022). However, the roles of N^6^-methyladenosine (m^6^A)-related lncRNA posttranscriptional modifications in CIRI remain unknown.

M^6^A is the most extensive modification of mRNA and ncRNA observed in eukaryotes, accounting for 80% of RNA methylation modifications, which can functionally regulate the transcriptome of eukaryotes, thereby affecting RNA splicing, nucleation, localization, translation, and stabilization (Meyer et al., 2012; Frye et al., 2018). M^6^A RNA methylation occurs in a variety of important cellular life processes, such as stem cell differentiation and the production of biological rhythms, and is also involved in the occurrence of various diseases (Dominissini et al., 2012). The process of methylation is reversible, and the level of RNA m^6^A methylation modification is regulated by methyltransferases and demethylases (Roundtree et al., 2017). Several current studies suggest that lncRNAs may regulate tumor growth through m^6^A modification in cancer (Steponaitis et al., 2022). Unfortunately, the role of m^6^A-related lncRNAs and mRNAs in CIRI has not been elucidated.

**FIGURE 1:**
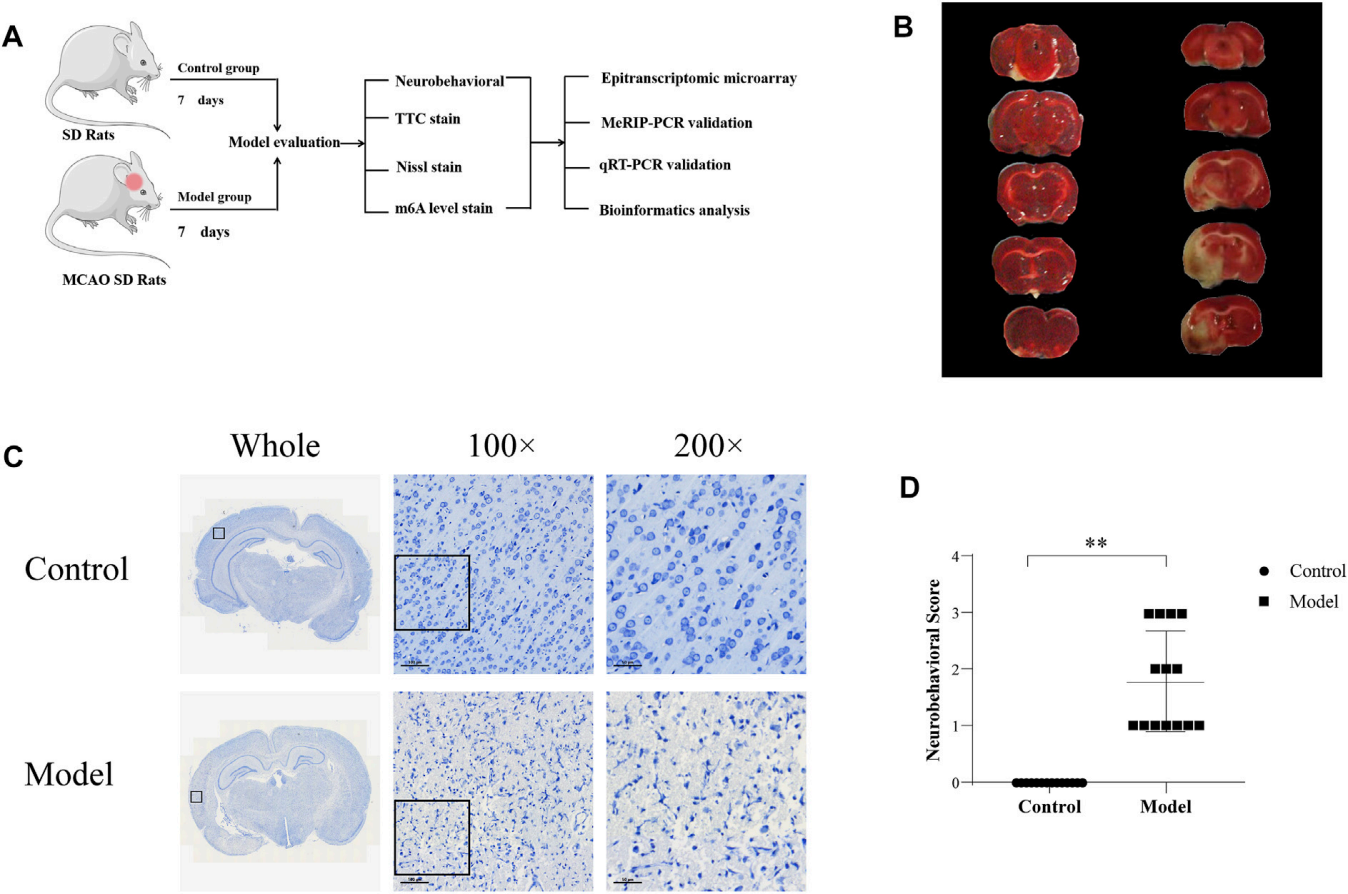
Establishment of the CIRI model **(A)** Experimental design **(B)** TTC detection (TTC staining displayed the infarcted areas in white and the normal areas in red) **(C)** Nissl staining **(D)** Neurobehavioral score.

In the present study, we identified the m^6^A lncRNA and mRNA modification profiles for the first time in CIRI. Bioinformatics analysis was used to predict the potential functions and related pathways of lncRNAs and mRNAs dysregulated by m^6^A modification in CIRI. Moreover, further analysis indicated that m^6^A modification of lncRNAs may exert biological functions through the lncRNA-miRNA-mRNA transcriptional network, as shown in Figure 1A.

## Materials and methods

### Animals

Specific pathogen-free (SPF) male Sprague Dawley (SD) rats that were 8 weeks old and weighed 250–260 g were purchased from Hunan Silaike Jingda Co., LTD (Changsha, China) using production license number SCXK (Xiang) 2019-0004. The animals were housed in the SPF animal room of the First Affiliated Hospital of Hunan University of Chinese Medicine at a temperature of 24–26°C, a relative humidity of 40%–60%, a day-night cycle of 12 h, and free access to food and water, and adaptive rearing was performed for 1 week before the experiment. This animal experiment was approved by the Experimental Animal Ethics Committee of the First Affiliated Hospital of Hunan University of Chinese Medicine (ZYFY20201215-1).

### Drugs and reagents

2,3,5-Triphenyl tetrazolium chloride (TTC) dye (Chengdu clone Chemicals Co., Ltd., CAS 298-96-4); arterial embolus (Beijing Cinontech CO., Ltd, 2636A2); RNA wait tissue preservation solution (Dalian Meilunbio Co., Ltd, MA0208); m^6^A RNA methylation assay kit (Abcam, ab185912); TRIzol reagent (Life Technologies, T9424); affinity-purified anti-m^6^A rabbit polyclonal antibody (Synaptic Systems, 202003); sheep anti-Rabbit IgG (Invitrogen, 11203D); and a lncRNA&mRNA epigenetic transcriptomic microarray (8 × 60 k) customized by Arraystar were used.

### Instruments

A centrifuge 5418 (Eppendorf, Germany); ultramicro spectrophotometer (Nanodrop, United States); bioanalyzer 2100 (Agilent, United States); G2505C microarray scanner (Agilent, United States); Vectra3 intelligent tissue slice imaging system (Perkin Elmer, United States); and Gene Amp PCR System 9700 (Applied Biosystems, United States) were used.

### Model preparation and grouping

Consistent with the method described previously, rats were randomly selected to establish the CIRI model (She et al., 2019): after the rats were anesthetized, an incision was made in the middle of the neck, the left internal and external carotid arteries and the common carotid arteries were exposed; the common carotid arteries were clipped to one small orifice, a wire plug was inserted until reaching the internal carotid artery at a depth of 2 mm from the bifurcation of the artery, and the internal carotid artery was ligated. Reperfusion was performed after the insertion of wire plugs for 2 h, and blood could re-enter the middle cerebral artery by the circle of Willis to achieve cerebral vascular reperfusion. The rats in the control group were operated on in the same way as the model group but were not given a plug line to occlude the middle cerebral artery. Finally, the wound was sutured, and the state of the rats was observed. The degree of neurological deficit in the rats was assessed by reference to the Zea-Longa scoring method (Longa et al., 1989), and a score of one–three points indicated successful modeling.

### Neurobehavioral score

Recent studies have indicated that the seventh day after cerebral ischemia is the optimal time for brain tissue recovery (Dos Santos et al., 2021). Therefore, in this study, the Longa 5-point scale was used to score neurobehavioral scores on the seventh day after CIRI in rats. The scoring criteria were as follows: 0 points, normal, no neurological signs; one point, the animal could not fully extend the left forelimb; two points, the animal’s left limb was paralyzed, the animal turned around to the left when walking, and tail-collision occurred; three points, the animal walked to the left and fell sideways, or the animal was unable to stand or roll; four points, no spontaneous movement, impaired consciousness.

### TTC detection

Consistent with the method described previously (Ni et al., 2022), after neurological evaluation, the rats were anesthetized; the whole brain was quickly removed; and the olfactory bulb, cerebellum, and lower brainstem were removed. The brains in each group were removed after being frozen in a freezer at -20°C for 15 min; placed on ice disks from the frontal pole to the occipital pole; centered at the level of the optic chiasm; and subjected to coronal, equal thickness sectioning with a slice thickness of 2 mm. The brain slices were immersed in 2% TTC staining solution and incubated at 37°C at constant temperature in the dark for 15 min. The stained hindbrain slices were placed in 10% formalin after fixation, removed, and blotted with filter paper to dry the surface moisture. The brain slices were arranged neatly in the anterior-posterior order of the brain, and a digital camera was used to obtain images.

### Nissl staining

After anesthetizing the rats, the whole brains were removed, fixed with 4% paraformaldehyde, and embedded in paraffin. The processed brain tissue was sectioned coronally and morphologically evaluated by Nissl staining. The sections were observed and photographed under a microscope.

### Quantification of total m^6^A levels in brain tissues

Total m^6^A levels were detected using a commercial m^6^A RNA methylation Quantification Kit. In brief, total RNA was first extracted using the TRIzol method, 200 ng of total RNA was added to each well, and reagents were added in steps according to the manufacturer’s instructions. Then, m^6^A levels were measured colorimetrically by reading the absorbance of each well at a wavelength of 450 nm.

### RT‒qPCR validation

The relative gene expression of m^6^A methylase was verified by RT‒qPCR. These genes included methyltransferases (writers), including methyltransferase like 3 (*METTL3*), methyltransferase like 14 (*METTL14*) and Wilms tumor one associated protein (*WTAP*), and demethylases (erasers), including fat mass and obesity-associated protein (*FTO*) and alkylation repair homolog 5 (*ALKBH5*). The experimental process strictly followed the steps of the kit. The internal reference gene was *β*-actin, and the 2^−ΔΔCt^ method was used to calculate the relative expression of the gene. The sequences of each primer are shown in Supplementary Table S1.

### Epigenetic transcriptomic microarray assays

The brains were decapitated directly after the rats were anesthetized, and the cortical tissue on the ischemic side was rapidly separated on ice, placed into a cryovial prefilled with RNA wait solution, stored strictly according to the operation procedure, and sent to Aksomics Biotechnology (Shanghai, China) for microarray assays. In brief, total RNA was first extracted using the TRIzol method, then the total RNA was immunoprecipitated with anti-m^6^A antibodies. The “IP” grade fraction of the immunoprecipitation was highly enriched for m^6^A methylated RNA, and the supernatant “Sup” grade contained unmodified RNA. The above two RNA types were amplified as cRNAs and mixed after labeling with Cy5 and Cy3. The samples were hybridized to the microarray for 17 h at 65°C in an Agilent Hybridization Oven. Slides were scanned with an Agilent G2505C microarray scanner. Data were extracted using Agilent feature extraction software. The generated raw data obtained from the files were normalized by GeneSpring software for subsequent data analysis.

### Gene ontology (GO) functional analysis and kyoto encyclopedia of genes and genomes (KEGG) pathway enrichment analysis

GO function and KEGG pathway enrichment analyses were performed on the associated mRNAs (Chen et al., 2022).

### MeRIP-PCR validation

Three lncRNAs (*LOC100912312*, *uc.440-* and *uc.77-*) and three mRNAs (protein phosphatase 1F (*PPM1F*), o-linked N-acetylglucosamine transferase (*OGT*) and schlafen family member 13 (*SLFN13*)) were randomly selected for validation. Total RNA was first immunoprecipitated with anti-m^6^A antibodies. The immunoprecipitated “IP” fraction contained enriched m^6^A methylated RNA, and the supernatant “Supernatant” fraction contained unmodified RNA. The above two kinds of RNA were converted into cDNA, amplified, and subsequently subjected to RT‒qPCR using gene-specific primers. In addition, MeRIP-PCR assays were performed in three replicates in each group (n = 3). The primer sequences are shown in Supplementary Table S2, and the proportion of m^6^A methylation modification of each gene was calculated according to the following formula.

$$\%Input=\frac{2^{-Ct\;MeRIP}}{2^{-Ct\;MeRIP}+2^{-Ct\;Supernatant}}\times 100\%$$ (1)

### Competing endogenous RNA (ceRNA) network establishment

We selected the above three validated lncRNAs for ceRNA network establishment. Similar to the results of a previous study (Chen et al., 2022), differentially methylated mRNAs with levels that were significantly positively correlated with lncRNA levels were first screened based on a Pearson coefficient >0.8. Then, miRBase and TargetScan were used to predict lncRNA‒miRNA relationship pairs. mRNA‒miRNA relationship pairs were predicted by the miRDB and miRWalk. Finally, a lncRNA‒miRNA-mRNA transcription network was established with miRNA as a bridge.

### Statistical analysis

Differentially expressed m^6^A methylation genes were screened by fold changes (FCs) of ≥1.5 and *p* values of <0.05. Measurement data are expressed as the mean ± standard deviation. If the data in each group conformed to a normal distribution, a one-way ANOVA was performed, and results with *p* values of <0.05 were considered statistically significant. Analysis was performed using GraphPad Prism 8 graphing software.

## Results

### Establishment of the CIRI model

TTC staining showed that the brain tissue in the control group was dark red, and no obvious pale cerebral infarction was found, while the model group showed obvious pale infarction, as shown in Figure 1B. Nissl staining showed a clear and complete neuronal cytoarchitecture in the cortical region of control rats, with uniform cytoplasmic and nuclear staining showing a pale blue color, normal intercellular spaces, and abundant numbers of Nissl bodies showing a dark blue color. Unlike in the control group, in the ischemic side of the cortex in the model group, neuronal cell body shrinkage was severe, vacuolar degeneration in the cytoplasm was obvious, cell arrangement lost regularity, and the number of Nissl bodies was reduced, as shown in Figure 1C. The neurobehavioral score of the rats in the model group was significantly higher than that observed in the control group (*p* < 0.01), as shown in Figure 1D. In conclusion, it was suggested that the rat model of CIRI was successfully established, and pathological damage was observed on the ischemic side of the rat model of cerebral ischemia.

### Detection of total m^6^A levels after CIRI

**FIGURE 2:**
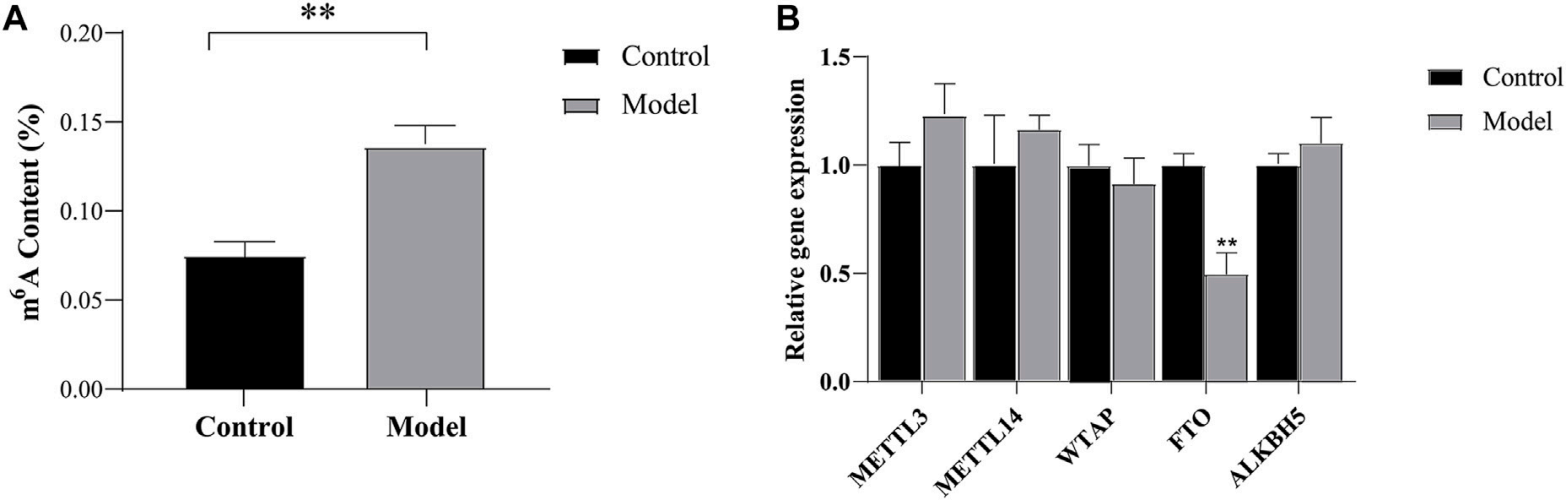
M^6^A modification and methyltransferases and demethylases validation **(A)** The total level of m^6^A modification measured in cortical tissue on the ischemic **(B)** Expression analysis of m^6^A methyltransferases and demethylases by RT-qPCR. ^**^ *p* < 0.01 vs Control group.

The results showed that the total m^6^A methylation level in the cortex tissue of the ischemic side was significantly higher in the model group than in the control group (*p* < 0.01), as shown in Figure 2A. This result suggested that CIRI resulted in the abnormal methylation of cortical tissue on the ischemic side.

### Verification of m^6^A methyltransferases and demethylases

We studied the expression levels of five m^6^A methyltransferases and demethylases and found that the expression of *FTO* was lower (*p* < 0.01) in the model group than in the control group, while no significant changes were observed in the expression of methyltransferases, including *METTL3, WTAP,* and *METTL14*. In addition, we also found no significant changes in the expression levels of *ALKBH5*, as shown in Figure 2B. These results suggest that the increase in total m^6^A levels in CIRI may be caused by the imbalance in the expression of *FTO*.

### M^6^A modification profiles of lncRNAs and mRNAs

**FIGURE 3:**
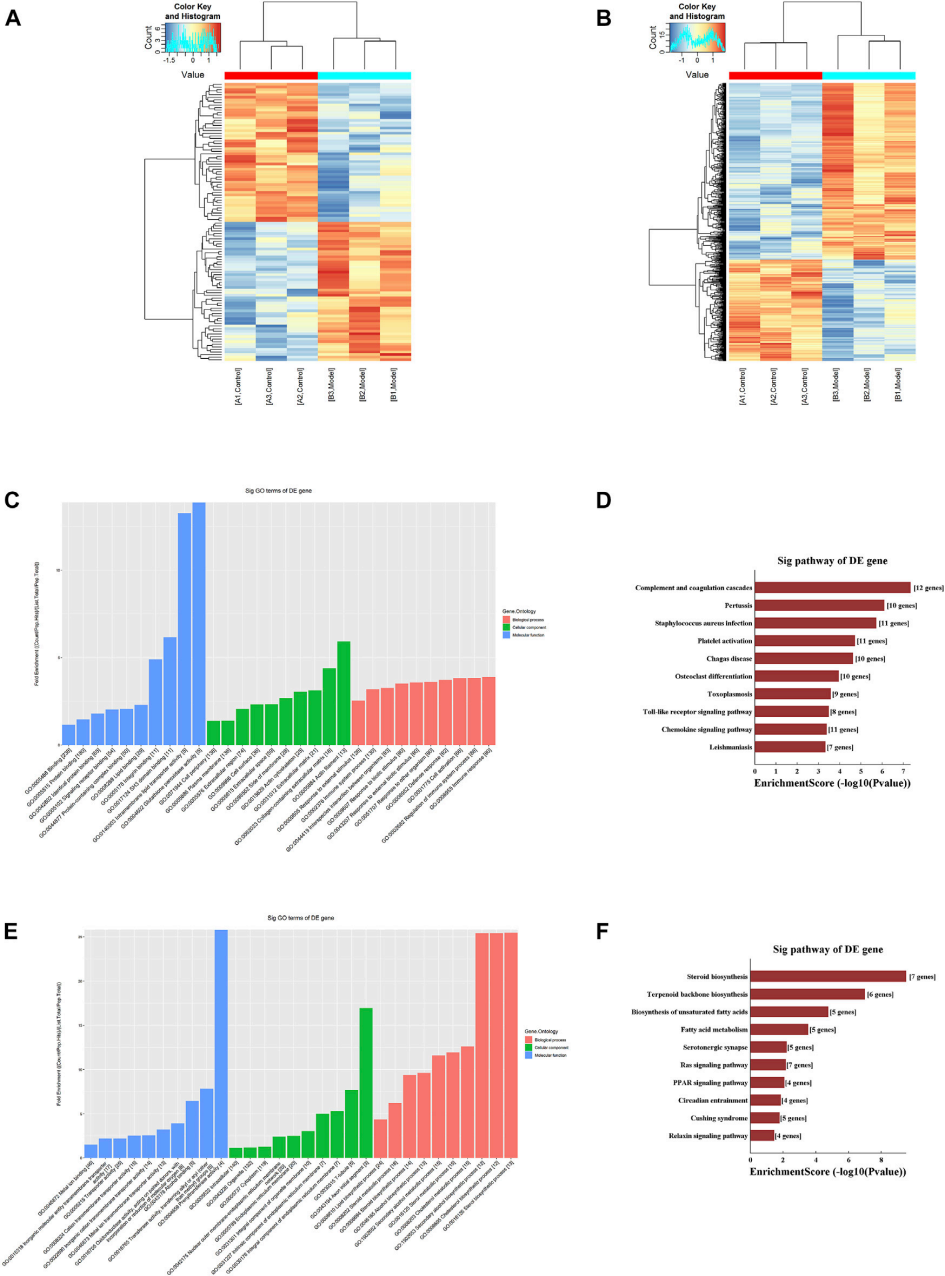
M^6^A modification profiles **(A)** Hierarchical clustering analysis of the differentially methylated lncRNAs **(B)** Hierarchical clustering analysis of the differentially methylated mRNAs **(C)** GO functional analysis of the hypermethylated mRNAs **(D)** KEGG pathway enrichment analysis of the hypermethylated mRNAs **(E)** GO functional analysis of the hypomethylated mRNAs **(F)** KEGG pathway enrichment analysis of the hypomethylated mRNAs.

Samples from the model and control groups were analyzed using m^6^A lncRNA and mRNA epigenetic transcriptomic microarrays. The results showed that a total of 108 lncRNAs exhibited differences in m^6^A modification between the model group and the control group, of which 54 were hypermethylated and 54 were hypomethylated, as shown in Figure 3A. In addition, 590 mRNAs harbored differential m^6^A modifications, of which 375 were hypermethylated and 215 were hypomethylated, as shown in Figure 3B. The raw data supporting this result have been uploaded to the GEO database (GSE201258).

### GO function and KEGG pathway enrichment analysis of differentially methylated mRNAs

First, we performed an enrichment analysis of the hypermethylated mRNAs and found that the biological processes involved were mainly protein binding, lipid binding, and glutathione peroxidase activity, and the cellular functions involved were mainly cell activation and immune response. The results of the KEGG pathway enrichment analysis mainly identified the chemokine signaling pathway and toll-like receptor signaling pathway, as shown in Figures 3C, D.

Moreover, we performed an enrichment analysis of the hypomethylated mRNAs and found that the biological processes involved were mainly ion binding and oxidoreductase activity, and the cellular components involved were mainly cell junctions, organelles, and synapses. The molecular functions involved mainly included various types of metabolism and biosynthesis. The results of the KEGG pathway enrichment analysis were mainly for steroid biosynthesis and the peroxisome proliferator-activated receptor (PPAR) signaling pathway, as shown in Figures 3E, F.

### Validation of differentially methylated genes

**FIGURE 4:**
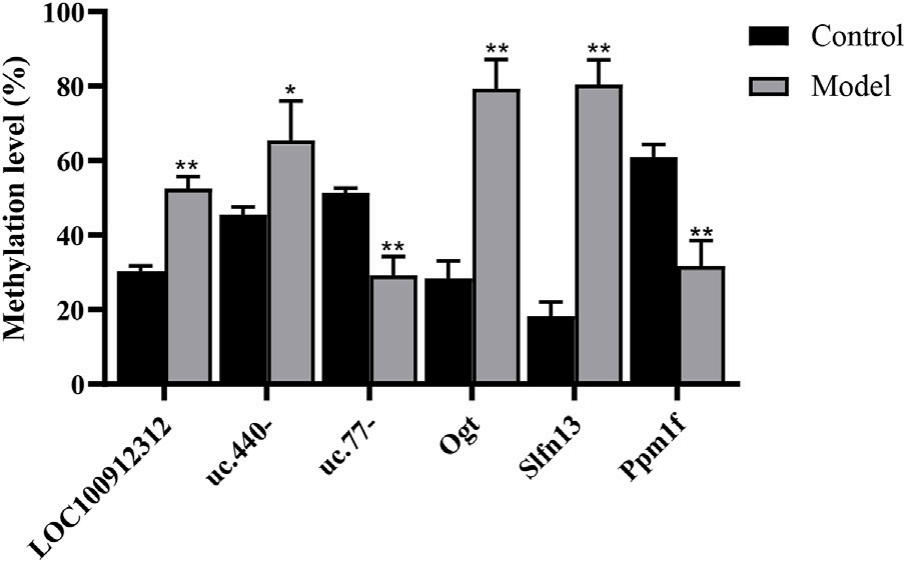
M^6^A analysis of differentially methylated genes by MeRIP-PCR. ^*^ *p* < 0.05, ^**^ *p* < 0.01 vs Control group.

Validation of the microarray results was performed by MeRIP-PCR. Three differentially methylated lncRNAs and three mRNAs were randomly picked. The results showed that in the model group, the m^6^A methylation ratios of *LOC100912312*, *uc.440-*, *OGT* and *SLFN13* were significantly increased (*p* < 0.05 or 0.01), and the m^6^A methylation ratios of *uc.77-* and *PPM1F* were significantly decreased (*p* < 0.01), consistent with the trends of change observed in the microarray results, as shown in Figure 4.

### CeRNA analysis of lncRNAs

**FIGURE 5:**
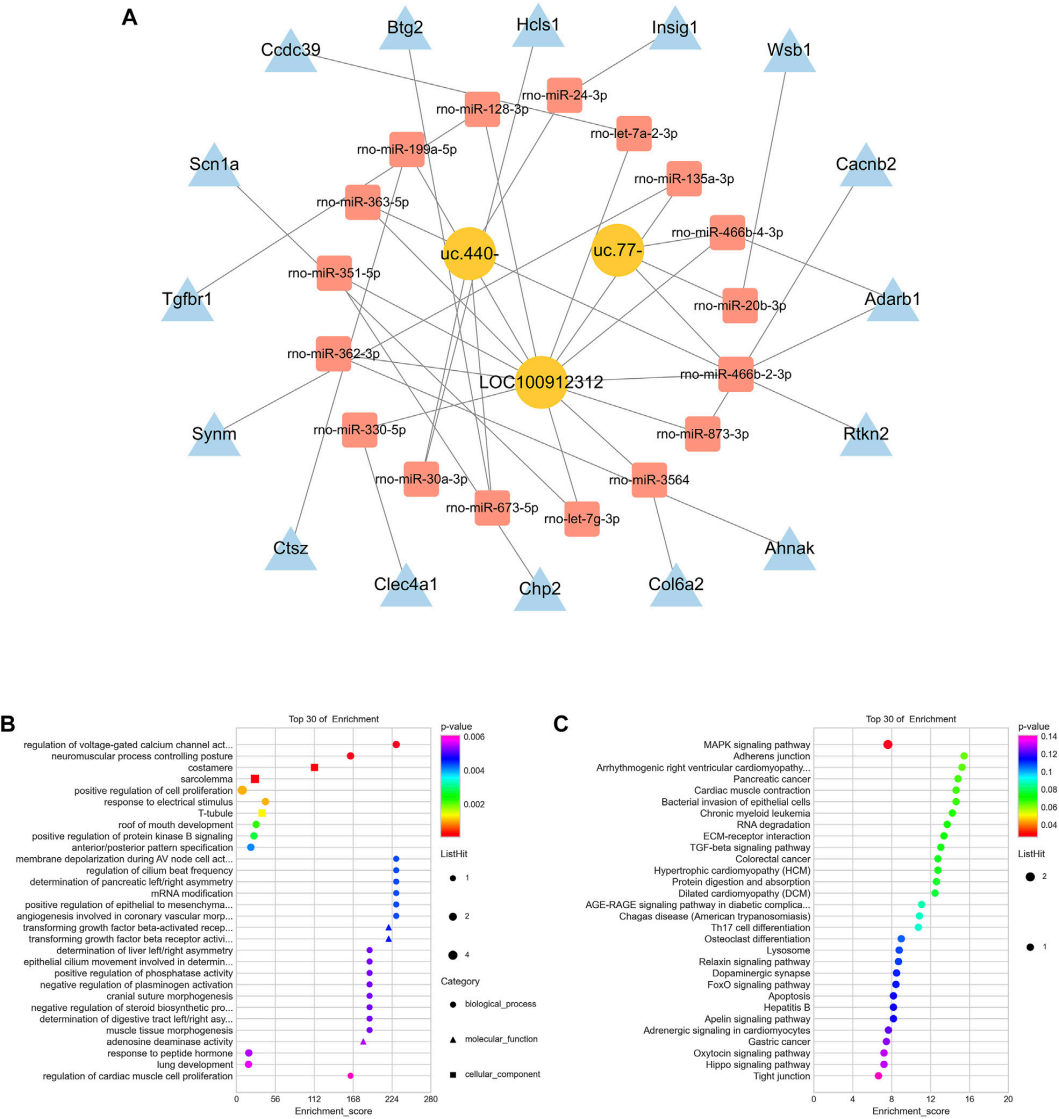
CeRNA analysis of lncRNAs **(A)** CeRNA network for *LOC100912312*, *uc.440-* and *uc.77-* **(B)** GO functional analysis involved in the ceRNA network **(C)** KEGG pathway enrichment analysis involved in the ceRNA network.

To clarify the biological function of related lncRNAs, we performed ceRNA network analysis on the validated *LOC100912312*, *uc.440-* and *uc.77-* lncRNAs based on the ceRNA hypothesis. A ceRNA network consisting of three lncRNAs, 17 miRNAs, and 16 mRNAs was established, as shown in Figure 5A. The enrichment analysis of mRNAs in this network showed that the biological processes were mainly involved in the regulation of voltage-gated calcium channel activity and neuromuscular process controlling posture, the cellular components were mainly involved in the costamere and sarcolemma, and the molecular functions were mainly involved in transforming growth factor beta-activated receptor activity (Figure 5B). The results of the KEGG pathway enrichment analysis mainly identified the mitogen-activated protein kinase (MAPK) signaling pathway, as shown in Figure 5C.

## Discussion

M^6^A is one of the most prevalent modifications present in the RNA of higher eukaryotes, and increasing amounts of evidence suggest that m^6^A modification of RNA plays important biological roles in physiological and pathological processes in the central nervous system (CNS) (Zhang et al., 2022). This finding was confirmed in cerebral ischemic disease, in which lncRNAs were identified as important biomarkers (Chokkalla et al., 2019; Xu et al., 2020a). Unfortunately, to date, reports on the dysregulation of lncRNA m^6^A modification in cerebral ischemia-reperfusion injury and studies on the biological functions of related lncRNAs have not been reported.

In this study, we first detected significantly elevated m^6^A levels in ischemic lateral brain tissue, which is consistent with the results of previous studies (Xu et al., 2020b). M^6^A modifications are known to be dynamic and reversible, installed by “writers” and removed by “erasers” (Shi et al., 2019). Therefore, we further investigated the expression levels of five common m^6^A methyltransferases and demethylases and found that *FTO* expression was downregulated after CIRI, while no significant changes were observed in the expression of methyltransferases, including *METTL3, WTAP,* and *METTL14*. The high expression of *METTL3*, *WTAP,* and *METTL14* may lead to an increase in the m^6^A methylation level (Zhang et al., 2022), but at present, there are few reports related to cerebral ischemia, which need to be further studied. *FTO* was initially considered related to obesity (Jia et al., 2011). With further research, *FTO* has been confirmed to be an important regulator of m^6^A methylation (Wei et al., 2018; Wei et al., 2022). *FTO* is an m^6^A demethylase with abundant expression in the brain (Li et al., 2018). Studies have shown that *FTO* expression is specifically downregulated in cerebral ischemic cortical neurons (Yi et al., 2021). In addition, the expression of *FTO* is downregulated in myocardial infarction (Mathiyalagan et al., 2019), suggesting that the expression of FTO is generally low in ischemic injury. Conversely, overexpression of *FTO* was shown to reverse m^6^A methylation and reduce the levels of neuronal apoptosis caused by cerebral ischemia (Xu et al., 2020b). Moreover, *FTO* has also been found to affect neurogenesis, memory formation, regulation of neuropsychiatric disorders, *etc.* (Li et al., 2017; Walters et al., 2017; Wang et al., 2022), suggesting that it may be closely related to the CNS. Moreover, we also found no significant changes in the expression levels of *ALKBH5*. Although *ALKBH5* has been reported to selectively demethylate *BCL2* transcripts after cerebral ischemia, which prevents degradation of B-cell lymphoma-2 (*BCL2*) mRNA, enhances expression of anti-apoptotic *BCL2* protein, and inhibits neuronal apoptosis, the role of *ALKBH5* is still less understood (Xu et al., 2020b).

Microarray analysis showed that 590 mRNAs exhibited differences in m^6^A modification in CIRI, of which 375 were hypermethylated and 215 were hypomethylated. Several studies have reported on the roles of related mRNAs. For instance, apolipoprotein E (*ApoE*) was recognized as a hypomethylated RNA in this study. In the CNS, *ApoE* is synthesized and secreted by astrocytes and is involved in maintaining the homeostasis of cholesterol and phospholipids, regulating the mobilization and redistribution of cholesterol and phospholipids during neural membrane remodeling and thus regulating the maintenance of synaptic plasticity as well as repair when neuronal cells are damaged (Cantuti-Castelvetri et al., 2018). In addition, vascular endothelial growth factor A (*VEGFA*) is recognized as a hypermethylated RNA; *VEGFA* is a factor that is closely related to angiogenesis after cerebral ischemia, and hypermethylated *VEGFA* can promote angiogenesis through multiple pathways (Xin et al., 2022). GO function and KEGG pathway enrichment analyses showed that these m^6^A-modified mRNAs were mainly involved in lipid binding, immune reactions, and oxidoreductase activity, and the signaling pathways involved were the Toll-like receptor signaling pathway and PPAR signaling pathway. The Toll-like receptor signaling pathway is a bridge between innate immunity and acquired immunity and has been confirmed to be closely related to the inflammatory cascade observed after cerebral ischemia (Eltzschig and Eckle, 2011). Proliferator-activated receptors (PARs) are ligand-activated receptors in the nuclear hormone receptor family that control metabolic processes in many cells. Studies have shown that ligand-activated PPAR can inhibit the expression of a variety of related inflammatory factors, thus inhibiting the inflammatory response and playing a protective role in CIRI (Wu et al., 2018). In addition, studies have supported the idea that PPAR activation has a direct transcriptional regulatory effect on the expression of several key endogenous antioxidants. For example, the activation of PPAR can activate the expression of antioxidant enzymes in female rats and protect them from CIRI (Mohagheghi et al., 2013).

In recent years, m^6^A-modified lncRNAs have received extensive attention. For example, m^6^A modification can drive the interaction between related lncRNAs and downstream genes, which indicates that the m^6^A modification status in lncRNAs may control the biological functions of lncRNAs (Lan et al., 2021). In this study, we identified 108 lncRNAs with differential m^6^A modification, of which 54 were hypermethylated and 54 were hypomethylated. Studies have found that although lncRNA cannot directly encode translational proteins, it can act as a “miRNA sponge”, indirectly reducing the binding between miRNA and downstream mRNA targets by absorbing the miRNA into this sponge, thus affecting the expression of target genes. This is essentially the mechanism underlying ceRNA regulation (Salmena et al., 2011). Subsequently, we established a potential ceRNA network of validated lncRNAs *LOC100912312*, *uc.440-,* and *uc.77-* to clarify the biological functions of relevant lncRNAs. Enrichment analysis indicated that this network was mainly closely related to the MAPK signaling pathway. MAPK signaling is an important regulatory pathway after cerebral ischemia and hypoxia and is closely related to pathological processes such as oxidative stress, the inflammatory response, apoptosis, and autophagy (Zhen et al., 2016). Experiments have confirmed that the activated MAPK pathway after cerebral ischemia can activate the NF-E2–related factor 2(Nrf2) pathway, and inhibiting the phosphorylation of the MAPK pathway can reverse the nuclear translocation of Nrf2 and reduce oxidative stress (Meng et al., 2018); other studies have indicated that the MAPK signaling pathway is associated with inflammatory responses. It can activate the downstream nuclear factor kappa-B (NF-κB) pathway after cerebral ischemia and promote the inflammatory response (Wang et al., 2014) while inhibiting the activation of the MAPK pathway can reduce the activation of the inflammatory response and have neuroprotective effects (Guo et al., 2012).

Undeniably, this study has several limitations. First, the sample size of this study was relatively small, and the above results may need to be validated in a large sample study in the future. In addition, targeting *FTO* in CIRI requires further exploration. Despite these limitations, we present the first systematic analysis of m^6^A lncRNA and m^6^A mRNA modification profiles in CIRI and constructed a related lncRNA‒miRNA‒mRNA network, laying a foundation for further revealing the pathogenesis of CIRI.

## Conclusion

In summary, we identified for the first time that m^6^A lncRNA and m^6^A mRNA were differentially modified in CIRI, and total m^6^A levels were increased in CIRI, which might be caused by the downregulation of *FTO* expression. In addition, bioinformatics analysis was used to predict the potential functions of differentially m^6^A-modified lncRNAs and m^6^A mRNAs, which could provide a reference for further revealing the mechanism of CIRI.

## Data Availability

The datasets presented in this study can be found in online repositories. The names of the repository/repositories and accession number(s) can be found below: https://www.ncbi.nlm.nih.gov; GSE201258.
